# Coexistence of a free-floating pigmented vitreous cyst and ciliary body cysts

**DOI:** 10.1186/s12886-026-05190-0

**Published:** 2026-08-07

**Authors:** Zhiyu Zhang, Yun Feng, Yan Yang

**Affiliations:** Department of Ophthalmology, Peking University First Hospital, Peking University, Beijing, 100034 China

**Keywords:** Pigmented vitreous cyst, Ciliary body cyst, Multimodal imaging, Ultrasound biomicroscopy, Fundus autofluorescence

## Abstract

**Background:**

Pigmented vitreous cysts are rare and generally benign intraocular lesions, but their origin remains uncertain. This case is noteworthy because multimodal imaging documented a free-floating pigmented vitreous cyst together with small bilateral temporal ciliary body cysts.

**Case presentation:**

A 32-year-old woman was referred after an incidental fundus finding in the left eye. She reported no visual symptoms, had a best-corrected visual acuity of 20/20 in both eyes, and had no relevant ocular history. Slit-lamp biomicroscopy revealed a free-floating, spherical, pigmented cyst in the left vitreous cavity that was markedly mobile with eye movements. B-scan ultrasonography demonstrated a 2.27 × 2.14 mm cystic lesion in the mid-to-posterior vitreous without retinal or choroidal attachment. Multimodal imaging, including ultra-widefield photography, fundus autofluorescence, swept-source optical coherence tomography, and ultrasound biomicroscopy, showed absent intrinsic autofluorescence of the cyst with blockage of background retinal autofluorescence and small bilateral temporal ciliary body cysts. Conservative observation was undertaken. At 2- and 7-month follow-up, the patient remained asymptomatic, with no apparent change in the clinical appearance of the cyst.

**Conclusions:**

Multimodal imaging can aid in the characterization of free-floating pigmented vitreous cysts and in the identification of associated anterior segment abnormalities. In this case, the coexistence of a vitreous cyst and bilateral temporal ciliary body cysts provides anatomic context for considering a possible displaced uveal pigment epithelial origin, although direct evidence of cyst detachment or migration was unavailable.

**Supplementary Information:**

The online version contains supplementary material available at https://doi.org/10.1186/s12886-026-05190-0.

## Introduction

Pigmented vitreous cysts are rare, generally benign intraocular lesions that are most often detected incidentally during routine ophthalmic examination or present with mild, intermittent visual disturbances [1, 2]. These cysts are typically free-floating within the vitreous cavity and may occur in either congenital or acquired settings [3].

Despite their characteristic clinical appearance, the origin of pigmented vitreous cysts remains incompletely understood. Displacement of pigment epithelial cysts arising from the iris or ciliary body has been proposed as a leading mechanism [4]. However, direct in vivo evidence supporting this hypothesis is limited [5]. Most published reports describe isolated vitreous cysts without associated anterior segment abnormalities, and systematic imaging evaluation of potential anterior segment sources has rarely been performed [6–10].

Herein, we report a case of a free-floating pigmented vitreous cyst coexisting with a ciliary body cyst in the same eye. Multimodal imaging was used to characterize both lesions and to explore their potential relationship, providing additional clinical insight into the possible origin of pigmented vitreous cysts.

## Case presentation

A 32-year-old woman was referred after an incidental abnormal fundus finding was detected in the left eye during a preoperative evaluation for refractive surgery at another institution. The patient reported no visual symptoms. Her medical and ocular histories were unremarkable, with no history of ocular trauma, inflammation, or intraocular surgery, and no relevant family history.

Best-corrected visual acuity was 20/20 in both eyes, and intraocular pressure was within normal limits bilaterally. Slit-lamp examination revealed no abnormalities in the anterior segment of either eye.

**Fig. 1:**
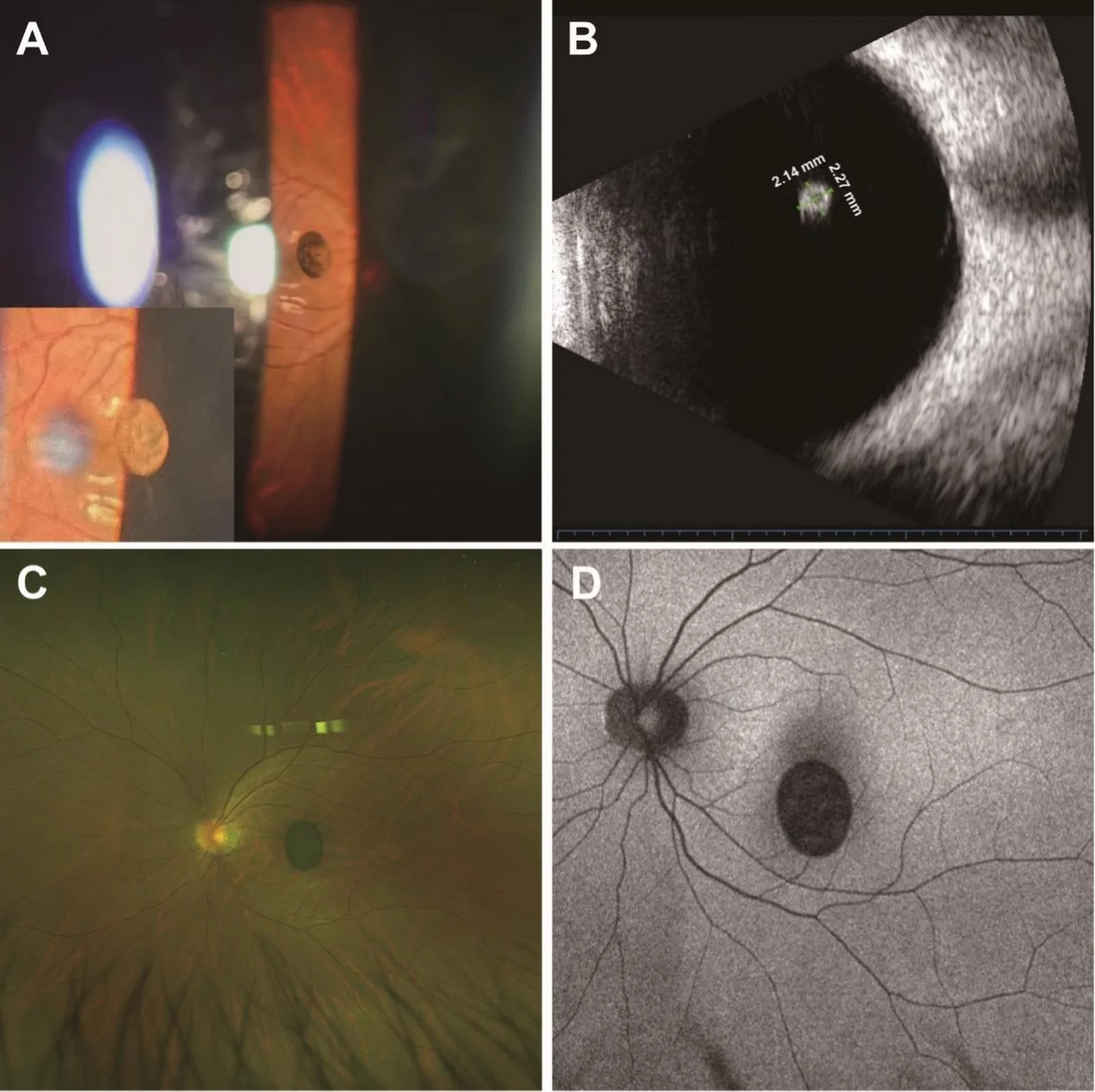
Multimodal imaging of the pigmented vitreous cyst in the left eye.** (A)** Indirect ophthalmoscope under a slit lamp biomicroscopy showing a free-floating pigmented cyst in the vitreous cavity. Inset: a separately acquired higher-magnification slit-lamp image of the same cyst. **(B)** B-scan ultrasonography demonstrating a cystic lesion in the mid-to-posterior vitreous cavity (2.27 × 2.14 mm) without retinal or choroidal attachment. **(C)** Ultra-widefield color fundus photograph demonstrating an approximately one–disc-diameter oval dark brown preretinal shadow covering the macular center. **(D)** Fundus autofluorescence imaging showing complete absence of autofluorescence within the cyst with blockage of background retinal autofluorescence

After pharmacologic pupil dilation, slit-lamp biomicroscopy revealed a free-floating, spherical cystic lesion within the vitreous cavity of the left eye. The lesion was well circumscribed, approximately one disc diameter in size, and covered by brown pigmentation. It demonstrated marked mobility with eye movements (Fig. 1A). The right eye showed no abnormal fundus findings.

B-scan ultrasonography of the left eye demonstrated a cystic lesion located in the mid-to-posterior vitreous cavity, measuring approximately 2.27 × 2.14 mm. The lesion had a well-defined hyperechoic wall and a hypoechoic internal cavity, without attachment to the retina or choroid, and without internal hyperreflective foci or calcification (Fig. 1B). Further multimodal imaging was performed. Ultra-widefield fundus photography, fundus autofluorescence (FAF), and swept-source optical coherence tomography (SS-OCT) were performed using the Optos Silverstone system (Optos). Ultra-widefield color fundus photography revealed a round, well-demarcated shadow anterior to the retina near the parafoveal region (Fig. 1C). FAF imaging showed complete absence of autofluorescence within the cyst, with corresponding blockage of background retinal autofluorescence (Fig. 1D).

**Fig. 2:**
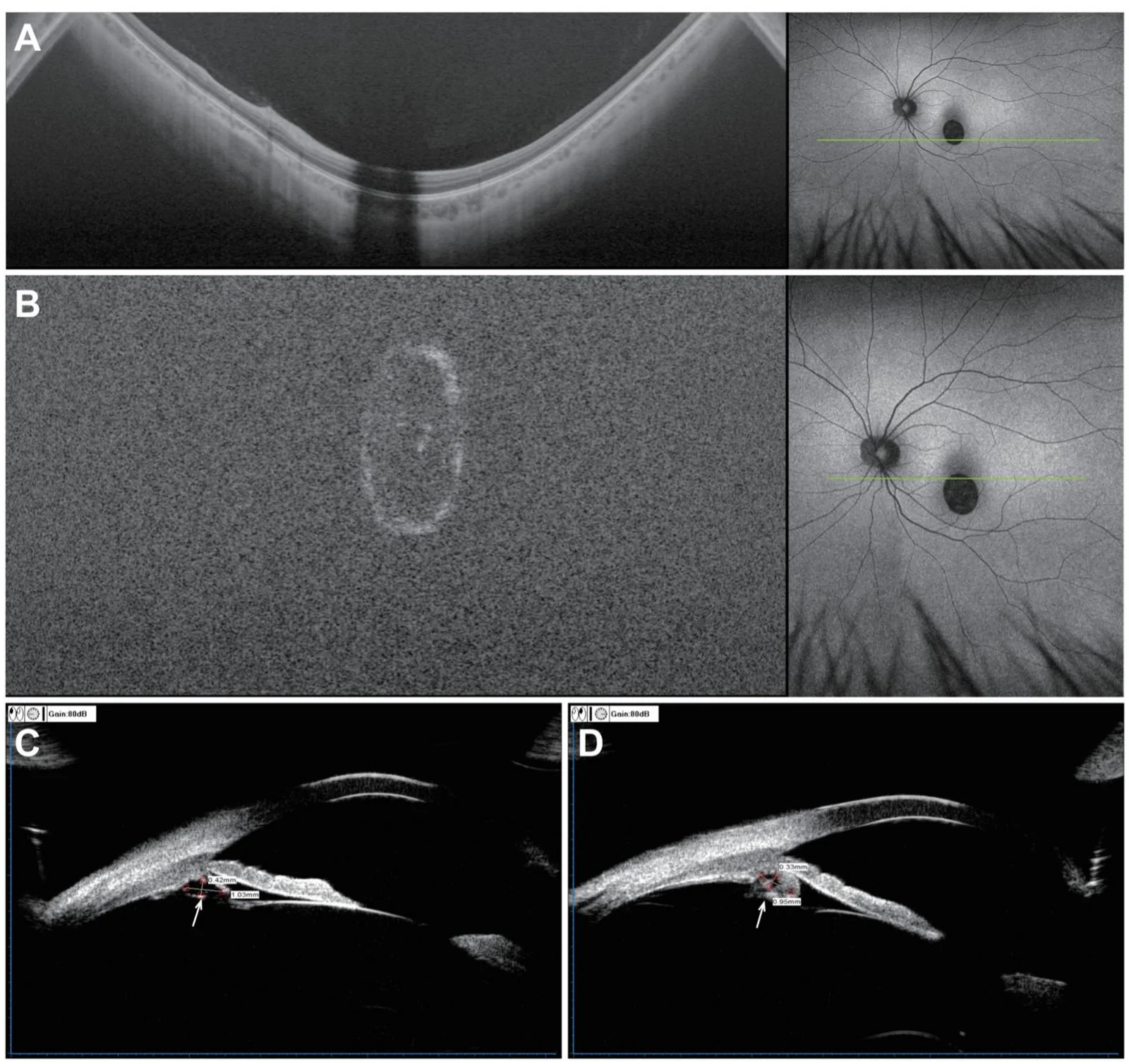
Swept-source optical coherence tomography (SS-OCT) and ultrasound biomicroscopy (UBM) findings**. (A)** SS-OCT B-scan of the left eye showing preserved retinal architecture with localized shadowing corresponding to the vitreous cyst. The scan location is indicated by the green line on the corresponding reference image. **(B)** SS-OCT image of the left eye obtained at a vitreous depth, demonstrating a cystic structure with a continuous hyperreflective wall and a hyporeflective internal cavity. The scan location is indicated by the green line on the corresponding reference image. **(C**,** D)** UBM showing focal hypoechoic cystic lesions in the right eye at approximately 9 o’clock (1.03 × 0.42 mm) and in the left eye at approximately 3 o’clock (0.95 × 0.33 mm), respectively. White arrows indicate the cystic lesions, and calipers show their approximate dimensions

SS-OCT demonstrated intact retinal architecture with localized shadowing corresponding to the vitreous cyst (Fig. 2A) and a cystic structure with a continuous hyperreflective wall and hyporeflective internal cavity (Fig. 2B). Although the cyst appeared spherical on clinical biomicroscopy, it appeared oval on the SS-OCT B-scan image. This discrepancy may reflect non-isotropic display scaling and aspect-ratio distortion.

Given the deep brown pigmentation of the vitreous cyst, which raised the possibility of a uveal pigment epithelial origin, Ultrasound biomicroscopy (UBM) was performed to evaluate the peripheral iris, iris pigment epithelium, ciliary body, and ciliary sulcus for clinically occult cystic or other abnormalities that might provide an anatomic clue to the lesion’s possible origin. UBM demonstrated small bilateral cystic lesions at the temporal iridociliary region. In the right eye, a focal hypoechoic cystic lesion at approximately 9 o’clock measured 1.03 × 0.42 mm; in the left eye, a corresponding lesion at approximately 3 o’clock measured 0.95 × 0.33 mm (Fig. 2C–D).

Given the absence of visual symptoms or functional impairment, the patient was counseled regarding the benign nature of the findings, and a conservative management strategy with regular follow-up was adopted. At 2 and 7 months, the patient remained asymptomatic, with no apparent clinical change in the cyst on slit-lamp biomicroscopy. Repeat UBM and other multimodal imaging were not performed. A dynamic clinical recording obtained at the 7-month visit provided qualitative documentation of the previously observed cyst mobility (Supplementary Video 1).

## Discussion

Pigmented vitreous cysts are uncommon intraocular findings and are generally regarded as benign. Although several pathogenetic mechanisms have been proposed, in vivo evidence supporting any single hypothesis is limited [11]. In this context, the present case is noteworthy for the coexistence of a free-floating pigmented vitreous cyst and small bilateral temporal ciliary body cysts documented by multimodal imaging. This combined finding provides an anatomic context for considering a possible uveal pigment epithelial source.

Two principal mechanisms have been proposed to explain the origin of pigmented vitreous cysts. Early histopathologic studies by Nork et al. [5] and by Lira et al. [12] demonstrated a single layer of pigmented epithelial cells with melanosomes and a basement membrane, findings interpreted as compatible with a developmental origin. In the absence of trauma, inflammation, or prior ocular surgery, this mechanism remains plausible. However, clinicopathologic evidence remains limited to a small number of surgically excised lesions, and clinical presentation alone does not reliably distinguish a developmental lesion from a detached pigment epithelial cyst.

Alternatively, pigmented vitreous cysts may arise from iris or ciliary body pigment epithelial cysts that become dislodged into the vitreous cavity [11]. Such cysts have been described in association with trauma, inflammation, retinal disorders, and prior retinal surgery, and may also occur as a result of mechanical stress related to ocular movements or intraocular pressure fluctuations, even in the absence of clinically apparent trauma or inflammation [13–15]. Orellana et al. [16] reported ultrastructural features resembling uveal pigment epithelium, and Patel et al. [17] described a surgically excised vitreous cyst with histopathologic features suggestive of iris pigment cells. Together, these findings support the biological plausibility of a uveal pigment epithelial mechanism, although the precise site of origin usually remains uncertain.

Multimodal imaging may provide additional in vivo clues. FAF imaging has been used to assess the presence of lipofuscin and thereby infer cellular origin. Lu et al. [7] reported a pigmented vitreous cyst without intrinsic autofluorescence, arguing against a retinal pigment epithelium source. UBM enables direct visualization of the ciliary body and peripheral anterior segment, facilitating the detection of pigment epithelial cysts that may otherwise remain clinically occult [18]. In the present case, absent intrinsic autofluorescence and small bilateral temporal ciliary body cysts provide anatomic context for a possible uveal pigment epithelial origin. To our knowledge, this is the first report in which FAF, SS-OCT, and UBM documented a free-floating pigmented vitreous cyst with ipsilateral temporal ciliary body cysts. These findings do not, however, identify the precise source lesion or directly demonstrate detachment or migration.

An important consideration is that ciliary body cysts may be incidental UBM findings in asymptomatic eyes. Kunimatsu et al. [19] detected cysts in 63 of 116 normal subjects (54.3%) and reported significant bilateral correlation, whereas Coc et al. [20] found cysts in 34.8% of a selected ICL-workup cohort. These prevalence data provide important context for interpreting the bilateral UBM findings in the present case. Although the observed temporal ciliary body cysts may be incidental and cannot be linked to a specific source lesion, their targeted detection provides an in vivo anatomic correlate for considering a possible uveal pigment epithelial mechanism of the pigmented vitreous cyst. The unilateral vitreous presentation may reflect a prior detachment or migration event from an occult peripheral lesion, although this possibility cannot be verified in this cross-sectional case [4].

From a clinical perspective, recognition of pigmented vitreous cysts is important to avoid misdiagnosis and unnecessary intervention. Multimodal imaging plays a central role in confirming the cystic and mobile nature of the lesion, excluding parasitic or neoplastic conditions, and identifying associated anterior segment abnormalities that may offer clues to its origin [21]. A free-floating vitreous cyst may mimic intraocular cysticercosis. Ganger et al. [22] reported an asymptomatic free-floating vitreous cyst initially referred as intravitreal cysticercosis, directly illustrating this diagnostic pitfall. Observation is appropriate for asymptomatic lesions, as most reported lesions appear stable over time [9]. Further longitudinal cases with standardized imaging are needed to clarify the natural history and potential clinical relevance of specific imaging phenotypes.

This case has several limitations. Histopathologic confirmation was unavailable, and no detachment or migration event was directly observed. Therefore, the precise tissue origin of the vitreous cyst could not be determined. In addition, UBM identified bilateral temporal ciliary body cysts, whereas the free-floating pigmented vitreous cyst was unilateral. A direct lesion-to-lesion relationship and causal association cannot be established from this single case.

## Conclusion

Multimodal imaging may provide valuable insight into the origin of pigmented vitreous cysts. The coexistence of vitreous and ciliary body cysts in this case provides an in vivo anatomic context for considering a possible uveal pigment epithelial origin and underscores the role of systematic imaging in accurate diagnosis and clinical management.

## Supplementary Information

Below is the link to the electronic supplementary material.

**Supplementary Material 1**: Supplementary Video 1: Dynamic clinical examination demonstrating free mobility of a pigmented vitreous cyst in the left eye with ocular movements.

## Data Availability

All data generated or analysed during this study are included in this published article and its supplementary information files.
